# Indirect Enzyme-Linked Immunosorbent Assay Based on Immunoglobulin Y Antibodies for the Evaluation of Humoral Immunity Against *Flavobacterium oreochromis* in *Colossoma macropomum*: A Preliminary Study

**DOI:** 10.3390/ani15060869

**Published:** 2025-03-18

**Authors:** Maria Vitória Lobo Corado, Elcimara Cardoso Pereira, Taísa Freitas Botinelly, Juliane Corrêa Glória, Rafael Luckwu de Sousa, André de Lima Guerra Corado, Antônio Alcirley da Silva Balieiro, Luís André Morais Mariúba, Silvia Umeda Gallani, Guilherme Campos Tavares

**Affiliations:** 1 Universidade Nilton Lins, Manaus 69058-030, AM, Brazil; mavimslobo@gmail.com (M.V.L.C.); elcimaracardoso94@gmail.com (E.C.P.); taisafbotinelly@gmail.com (T.F.B.); andre.corado@uniniltonlins.edu.br (A.d.L.G.C.); silviaugallani@gmail.com (S.U.G.); 2 Instituto Leônidas e Maria Deane, Fiocruz Amazônia, Manaus 69057-070, AM, Brazil; juliane.gloria@fiocruz.br (J.C.G.); alcirley@gmail.com (A.A.d.S.B.); andre.mariuba@fiocruz.br (L.A.M.M.); 3 Programa de Pós-Graduação em Biologia da Interação Patógeno Hospedeiro, Instituto Leônidas e Maria Deane, Fiocruz Amazônia, Manaus 69057-070, AM, Brazil; 4 Programa de Pós-Graduação em Imunologia Básica e Aplicada, Universidade Federal do Amazonas, Manaus 69057-070, AM, Brazil; rafalucksousa@gmail.com; 5 Programa de Pós-Graduação em Biotecnologia, Universidade Federal do Amazonas, Manaus 69067-005, AM, Brazil; 6 Departamento de Medicina Veterinária Preventiva, Escola de Veterinária, Universidade Federal de Minas Gerais, Belo Horizonte 31270-901, MG, Brazil

**Keywords:** IgY, immunoassay, plasma, tambaqui, vaccine

## Abstract

Infections with *Flavobacterium oreochromis* pose risks to the production of *Colossoma macropomum*. Since no therapeutic treatment exists for this disease in this aquatic host, prophylaxis through vaccination is one method for disease control. The enzyme-linked immunosorbent assay (ELISA) is widely used in aquaculture to assess animal immunity and, mainly, to validate vaccination protocols. The method based on the use of immunoglobulin Y (IgY) is an alternative to mammalian antibodies that should be considered due to its advantages over antibodies produced by small mammals. This study aimed to standardize an indirect ELISA (iELISA) protocol to validate the immunization of *C. macropomum* against *F. oreochromis*. For this, a hen and fish were immunized to obtain anti-*C. macropomum* and anti-*F. oreochromis* antibodies, respectively, which were used to standardize the test. Fish were inoculated with bacterin + adjuvant, bacterin alone, adjuvant alone, or phosphate-buffered saline, subjected to blood sampling, and monitored for up to 60 days. The iELISA was composed of the inactivated bacteria in the coating step, *C. macropomum* anti-*Flavobacterium oreochromis* as the primary antibody, and anti-*C. macropomum* IgY as the secondary antibody. The results demonstrate that the highest antibody titers were found in the organisms immunized with bacterin + adjuvant and bacterin. The iELISA enabled the detection of antibodies produced by *C. macropomum*, which were mediated by IgY specificity.

## Simple Summary

Columnaris-causing bacteria drastically affect tropical freshwater fish, including *Colossoma macropomum*. High mortality rates associated with columnaris have been reported in both natural and experimental infections. To develop vaccination protocols for this disease, it is necessary to validate the immunological memory against the target pathogen through laboratory analyses, such as the indirect enzyme-linked immunosorbent assay (iELISA). Here, we aimed to standardize an iELISA using IgY extracted from chicken plasma to validate an experimental vaccination protocol applied to *C. macropomum* against *Flavobacterium oreochromis*. Our results demonstrated that the IgY obtained from hen serum was able to react specifically with epitopes present in the serological material of *C. macropomum* and can be utilized in the standardized iELISA protocol to detect antibodies from this fish species immunized against *F. oreochromis*.

## 1. Introduction

*Colossoma macropomum* is a native freshwater fish species with significant economic value in Latin America, particularly in Brazil [1]. In this country, the species is the second most produced among cultivated fish species, reaching a volume of 113.6 thousand tons in 2023 [2], and the fourth most exported, with a volume of 226 tons in 2024 [3]. Despite this, the species faces challenges in the intensification of its production. Among the major issues related to the production of this fish species, bacterial diseases can be highlighted, with infections caused by *Aeromonas hydrophila* [4], *Aeromonas jandaei* [5], *Edwardsiella tarda* [6], and *Flavobacterium oreochromis* [5] having already been reported.

Columnaris-causing bacteria drastically affect tropical freshwater fish like *C. macropomum* [7,8,9]. These bacteria reside in water, ponds, mud, and sediments and can survive for extended periods in various environmental conditions [10]. Fish affected by these pathogens may present clinical signs such as anorexia, lethargy, erratic swimming, rapid opercular movements, gill lesions, tegument erosion, fin erosion, melanosis, and saddleback lesions [5]. High mortality rates associated with columnaris disease have been reported in both natural and experimental infections [8,11]; however, vaccines (formalin-killed whole cells, formalin-killed sonicated cells, and mucoadhesive nano-encapsulated vaccines) have been developed as a prophylactic method against *F. oreochromis* [12]. Through vaccination, the immune system is stimulated to develop memory antibodies, thus enhancing the acquired immune response upon exposure to the pathogen [13].

To develop vaccination protocols, it is necessary to validate the immunological memory against the target pathogen through laboratory analyses such as the indirect enzyme-linked immunosorbent assay (iELISA). This method involves coating a microplate with an antigen, followed by the sequential addition of a primary antibody and a secondary antibody linked to a revealing enzyme [14]. The ELISA requires specific antibodies for its standardization, which are used as biotechnological tools [15]. Immunoglobulin G (IgG) produced by mammals is widely used in research and laboratory diagnostics, but its extraction is invasive, often involving bloodletting and euthanasia of animals like mice and rabbits to obtain large blood volumes [16]. As an alternative to IgG, immunoglobulin Y (IgY), which is produced by chickens, provides immune support during the embryonic and post-natal phases [17]. IgY is abundantly produced in the egg yolk and serum of birds, can be generated rapidly with minimal environmental impact, requires low investment in infrastructure, and has low production and handling costs when obtained from chickens [18]. IgY is phylogenetically and structurally distinct from IgG, which reduces the likelihood of the cross-reactivity of this immunoglobulin with IgG receptors in mammals, as well as background noise and false-positive results [19]. Furthermore, IgY has been used in immunoassays as a secondary antibody (an antibody prepared in a different host), as it facilitates cellular-level detection and binding with different immunoglobulin subtypes [20]. IgY has been used in aquaculture with good results for diagnostic purposes in various aquatic animals [21] and has already been applied in immunological assays for the detection of infection by *Aeromonas hydrophila* in fish [22].

Combining the potential use of IgY with emerging vaccinations against columnaris and considering the scarcity of information on the immunity and antibody titers in *C. macropomum*, this study aimed to standardize an iELISA using IgY extracted from chicken plasma to validate an experimental vaccination protocol applied to *C. macropomum* against *F. oreochromis*.

## 2. Materials and Methods

### 2.1. Hen Immunization

For the immunization of the hen with *C. macropomum* immunoglobulins, they were first extracted using the protocol described by Dias et al. [23]. In summary, *C. macropomum* blood was collected from the caudal vein and placed in tubes containing heparin (Liquemine, Roche™, Rio de Janeiro, Brazil), and then the samples were centrifuged at 2000× *g* at 25 °C for 5 min to obtain the plasma. The plasma sample had its pH adjusted to 5 with acetic acid, and 8.7% of the final volume of caprylic acid was added, which was incubated under agitation for 30 min. After incubation, the sample was centrifuged at 21,697× *g* for 15 min, and the supernatant was recovered and filtered with a 0.45 µm membrane (Millipore Corporation, Burlington, MA, USA).

A 71-week-old Dekalb White laying hen (1725 g) was acquired from a commercial laying farm and maintained in an individual cage with drinking water and food provided ad libitum. The hen was immunized via multiple pectoral muscle injections on days 0, 14, and 21 with an inoculum containing 9.11 μL of *C. macropomum* immunoglobulin extract purified with caprylic acid [23] and 300 μL of Montanide™ ISA adjuvant (Seppic, Paris, France), which was topped up to 600 μL with phosphate-buffered saline (1× PBS, pH 7.2). Immunizations were carried out by the Ezscience company (Manaus, Brazil). Blood samples (1.0 mL) were collected from the hen’s ulnar vein on days 0, 7, 14, and 21 and immediately transferred to 1.5 mL tubes without an anticoagulant. The aliquots were kept in the refrigerator for 2 h and then centrifuged at 2000× *g* at 4 °C for 12 min. The obtained plasma was stored at −80 °C until use in the ELISA standardization.

### 2.2. Bacterial Inoculum and Immunization of Fish Against Flavobacterium oreochromis

The *Flavobacterium oreochromis* strain (AM-FO14) was previously obtained from diseased *Colossoma macropomum* and ascertained according to pathogenicity (complete Koch’s Postulate) and identity (PCR-multiplex, REP-PCR, and complete genome sequencing) [24]. The strain (stored at −80 °C in G broth enriched with glycerol 15%) was thawed and streaked onto G agar at 28 °C for 48 h. A colony was picked, inoculated into G broth, and incubated under the same conditions, at 100 rpm. The bacteria were resuspended in sterile 1× PBS at a concentration of 3.3 × 10^9^ colony-forming units (CFU)/mL, which were determined using the plating-count method [25]. The bacterial inoculum was inactivated with 1% formalin and incubated at 4 °C for 24 h [26]. Bacterin synthesis was confirmed by the absence of bacterial growth on G agar (28 °C for 48 h). To produce the oil-adjuvanted vaccine, antigen (bacterin) and adjuvant (Montanide™, Seppic, Paris, France) were mixed at a ratio of 27:73 (*v*/*v*), respectively [27], and emulsified.

For the immunization, fifty-four juvenile *C. macropomum* with no history of diseases were acquired from a population maintained in tanks at Nilton Lins University. Prior to the experimental assay, six fish were randomly selected and submitted to bacteriological examination to ensure they were free from bacterial infections. The remaining fish were acclimated for 10 days and later randomly distributed into four groups with two replicates each: G1—group inoculated with bacterin + adjuvant, G2—group inoculated with bacterin, G3—group inoculated with adjuvant, and G4—control group (inoculated with sterile 1× PBS). The fish (45.5 ± 9.5 g) were kept in polyethylene tanks (80 L) at a density of 6 fish per tank (0.075 fish/L), with a supplementary aeration system, daily partial water exchange at a rate of 70%, and a temperature of 28 °C. Commercial fish feed (Nutripiscis, Brazil, 36% protein) was provided twice per day (2% body weight daily) throughout the experimental and maintenance period. Immunization was carried out on days 0 and 21 via intraperitoneal injection (i.p.), after anesthesia (benzocaine, 100 mg/L, immersion bath), with 0.2 mL of inoculum per fish. On day 30, the experimental groups were inoculated with bacterin via i.p. (0.1 mL of the inoculum for each 10 g of live weight, 10^9^ CFU/mL [5]) to stimulate the humoral response, except G4, which was inoculated with sterile 1× PBS. In order to evaluate humoral responses, blood samples (0.5 mL) were collected from all fish via puncture of the caudal vein after anesthesia at 0, 21, 30, 40, 50, and 60 days post-inoculation (dpi) and immediately transferred to 1.5 mL tubes without an anticoagulant. The collected blood was kept in a refrigerator for 2 h, followed by centrifugation at 2000× *g* at 4 °C for 12 min to separate the serum and plasma from the blood. Aliquots of plasma were collected, transferred to another tube (1.5 mL), and stored at −80 °C until use in the standardization of the ELISA.

### 2.3. Standardization of the Indirect Enzyme-Linked Immunosorbent Assay (iELISA)

For the standardization of the iELISA, polyethylene microplates (Kasvi, Pinhais, Brazil) were coated with inactivated *F. oreochromis* diluted in carbonate/bicarbonate buffer (1:1000). A volume of 50 µL of concentrated antigen (0.5 × 10^2^ CFU/mL) was added per well, and the microplates were incubated in a humid chamber for 1 h at 37 °C. For blocking, 150 µL of ELISA Blocking Buffer (Scienco Biotech, Lages, Brazil) was added to each well, followed by incubation under the same conditions described above. Next, the plate was washed four times with 200 µL per well of 1× PBS with 0.05% Tween-80 (PBST). After the washes, 50 µL of the primary antibody (anti-*F. oreochromis* from fish) was tested at the following dilutions: 1:50, 1:100, 1:200, and 1:400. Then, the blocking buffer was added to each well and incubated at 37 °C for 1 h. Five further washes with 200 µL PBST were performed. Then, 50 µL of the unmarked secondary antibody (anti-*C. macropomum* IgY), diluted at 1:50, 1:100 or 1:150 in ELISA blocking buffer was added in each well to recognize the primary antibody bound to the antigen fixed to the plate. The plate was again incubated in a humid chamber at 37 °C for 1 h, followed by five washes with PBST. Then, 50 µL of the labeled secondary antibody, goat anti-chicken IgY (Sigma-Aldrich, Darmstadt, Germany, catalog number: SAB3700226), diluted at 1:10,000 in ELISA blocking buffer, was added to each well, followed by incubation (37 °C for 1 h) and five washes with PBST. The reaction step was carried out using One Step—TMB Linear (Scienco) chromogenic substrate, added at 50 µL/well, with a 2 min reaction time. The reaction was stopped with 25 µL of 1 M sulfuric acid in each well. The plates were read in an ELISA plate reader (LMR-96, Loccus, Cotia, Brazil) at a wavelength of 450 nm to assess optical density. All measurements of the samples were tested in triplicate.

### 2.4. Statistical Analysis

The ideal dilution of the primary and secondary antibodies was the one that showed the greatest difference in optical density between positive (G1 and G2) and negative (G3 and G4) samples. After standardizing the technique, the comparison between experimental groups was performed using linear mixed models, which were implemented according to the experiment’s longitudinal dependency. The cut-off point was defined using the mean optical density readings from negative samples multiplied by three times the standard deviation [28]. Groups with antibody levels equal to or greater than the cut-off were considered immunized. Those with lower results were considered unimmunized. Statistical analysis was conducted using R software v.4.3.1 [29], with various packages (tidyverse v.2.0.0, lme4 v.1.1.36, sjPlot v.2.8.17, and hnp v.1.2.6), with a significance level of *p* < 0.05.

## 3. Results

### 3.1. Immunized Animals

According to the observation and monitoring after restraint and immunization, no behavioral or clinical changes were observed in the hen immunized with *C. macropomum* immunoglobulin. In the fish, lethargy and partial loss of appetite were observed only after the first 24 h following handling, anesthesia, and immunization in all the tested groups. No further changes were noted thereafter.

### 3.2. Standardized iELISA and Analysis

**Figure 1:**
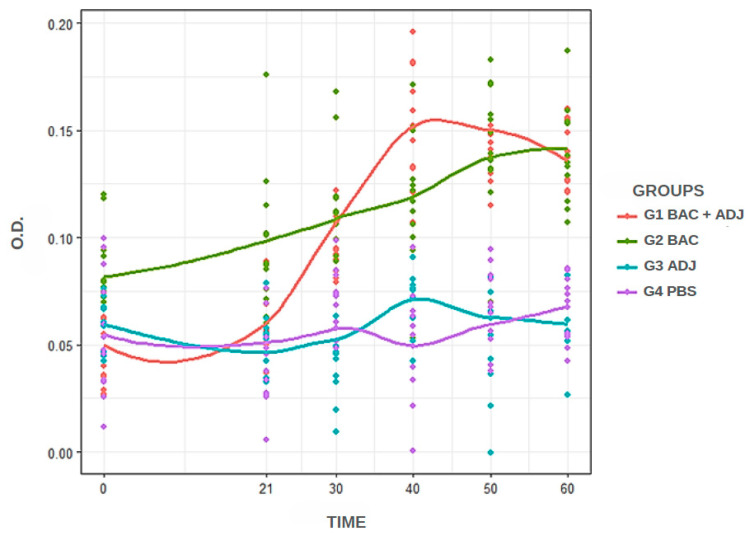
Dynamic changes in antibody production of immunized animals in each group over time at 0, 21, 30, 40, 50, and 60 days. Similar results were observed between G1 and G2, with differences compared to G3 and G4.

The standardized iELISA was performed under the following conditions: 50 μL of antigen per well, 50 μL of *C. macropomum* plasma diluted at 1:400 (best dilution of the primary antibody), 50 μL of anti-*C. macropomum* IgY at 1:100 as the unlabeled secondary antibody, and 50 μL of anti-IgY at 1:10,000 as the labeled secondary antibody. Under these conditions, the temporal samples from the experimental groups were subjected to absorbance testing and analysis. Figure 1 shows the distribution of antibody titers among the experimental groups. In general, groups G1 and G2 exhibited higher antibody titer values, while the groups injected with adjuvant (G3) and PBS (G4) inoculum showed lower titer values. When analyzing absorbance over time, it is evident that the G2 group had a stronger reaction at day 0 compared to the other groups, but it was surpassed by the G1 at 40 and 50 days. The G1, G3, and G4 groups showed similar titration levels at 0 and 21 days, but G1 increased antibody titers from day 30, the day on which the fish were re-inoculated with bacterin.

From the linear regression (mixed model) analysis, it was possible to statistically compare G1 in relation to the other groups (Table 1). The analysis showed that G1 and G2 were similar, and both differed from groups G3 and G4.

**Figure 2:**
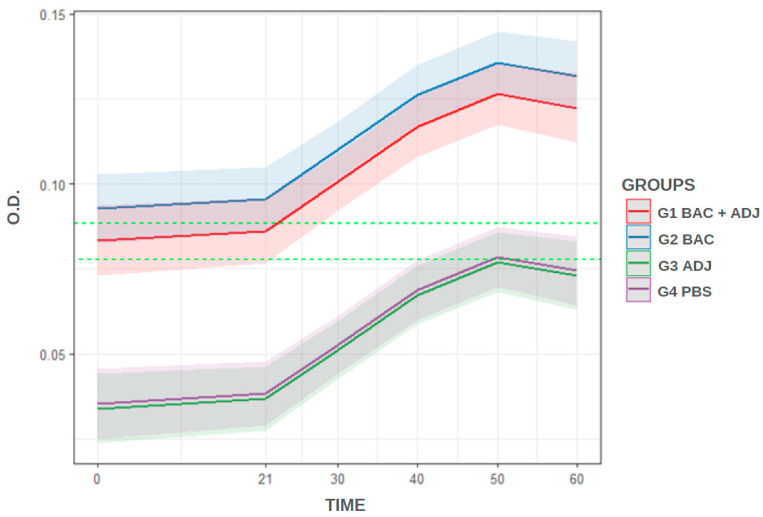
Representation of the model’s prediction. Cut-off determined at 0.088 (3× standard deviation, upper dashed line) and 0.077 (2× standard deviation, lower dashed line) OD_450_.

According to the cut-off that was determined (0.088 OD_450_), G1 and G2 were considered immunized from 21 dpi (exceeded cut-off value). Groups G3 and G4 did not reach the cut-off throughout the analysis and are therefore considered unimmunized. The model’s prediction (Figure 2) shows the overlap of confidence bands between G1 and G2, demonstrating the statistical similarity between those immunized with bacterin + adjuvant and bacterin. The figure also shows the overlap of bands between G3 and G4, demonstrating the statistical similarity between those that are unimmunized. According to the regression analysis (Table 1), the immunized groups differ (*p* < 0.05) from unimmunized groups.

## 4. Discussion

Humoral immunity encompasses the antibody-mediated response and is an essential factor to explore, given the importance of B lymphocytes and plasma cells in producing immunological memory, which provides the host with extensive protection against aggressive and harmful agents [30]. In view of this, diagnostic methods such as ELISA, which favor the monitoring of animal health and immune response after vaccination protocols, are being implemented [21,31,32,33]. ELISA is a methodology that has been applied in aquaculture to detect and quantify pathogens and, through serological samples, can measure the host’s antibody-mediated response [34]. In addition, the technique has been used in the study of fish food safety to evaluate the presence of allergens [35], toxins in fish [36], and even the bioaccumulation of substances such as antimicrobials [37]. These factors, combined with the high accuracy of the test, optimization of protocol execution, and low cost of the analysis [38], make it an effective tool for validating vaccine protocols through the measurement of specific antibody expression [33]. For example, Wang et al. [39] evaluated the antibodies produced by *Mylopharyngodon piceus* after vaccination against *Aeromonas hydrophila* using a precise ELISA protocol to measure the titration of specific antibodies against the bacteria. In *Lota lota*, the measurement of antibodies against *Aeromonas* sp. was performed after a bacterial challenge [40]. In addition, iELISA has already been established to evaluate humoral immunity in fish species such as *Ictalurus punctatus* [41], *Oreochromis niloticus* [42], and *Piaractus mesopotamicus* [43]. Despite the imminent risks that *Flavobacterium* sp. can pose to fish production, to our knowledge, to date, there is no description of this fundamental tool for the detection of antibodies against *Flavobacterium oreochromis* that infects *C. macropomum*.

For the standardization of the iELISA, the implementation of IgY technology is an ethical and cost-effective alternative to mammalian antibodies [44]. In the 1990s, IgY antibodies were internationally recognized as an alternative method for producing polyclonal antibodies when the scientific community’s ethical premise on animal welfare was also established [45]. Their practical relevance has attracted interest in both basic and applied research [46] due to the abundant production of polyclonal antibodies in chicken plasma and egg yolk [47]. Unlike the production of antibodies in mammals, the use of IgY eliminates the need for bleeding and killing animals and produces a high yield of antibodies [48]. Additionally, it offers practical material collection and ease of handling [49], especially when extracted from chicken eggs. In aquatic animal health studies, IgY has been used as an alternative method for treating infectious diseases [50] such as *Edwardsiella tarda* in *Scophthalmus maximus* [51], herpesviral hematopoietic necrosis in *Carassius gibelio* [52], *Aeromonas hydrophila* in *Carassius auratus* [53], and *Vibrio harveyi* in *Takifugu rubripe* [54].

IgY is also a valuable tool in biotechnology and has excellent performance when applied in immunodiagnosis [55]. The use of IgY has already been described for the immunodetection of *Aeromonas hydrophila* [56] and *Streptococcus agalactiae* [57] in *Oreochromis niloticus*. In this aquatic species, tests using IgY have been reported as being effective for diagnosing infectious conditions, such as endocarditis and bacterial meningitis caused by *Streptococcus agalactiae*, through the intracellular and extracellular detection of the bacteria in tissues via immunofluorescence and immunohistochemistry analyses [57]. Additionally, *Aeromonas hydrophila* was detected in phagocytic cells and in the tissues of *Oreochromis niloticus* using IgY (Fernandes et al., 2019) [56]. To date, among Brazilian native fish species, there are records of the implementation of IgY biotechnology only in *Piaractus mesopotamicus*. Fernandes et al. [22] evaluated the potential of IgY as a tool in immunohistochemistry tests for the detection of *Aeromonas hydrophila*. Specific IgY has demonstrated high specificity for bacterial epitopes, cytoplasm, and exotoxins, enhancing the capacity for diagnosis of bacteriosis at different stages of the infection [22]. Despite evidence of promising applicability in fish farming, to our knowledge, this study is the first to implement a methodology for IgY production using an Amazonian fish species.

The standardization of the iELISA developed in this study demonstrated that IgY specific to *C. macropomum* (anti-*C. macropomum* IgY) was successfully recognized by the fish serum, indicating that the immune response or the presence of the target protein in the serum can be successfully detected by IgY antibodies. Therefore, it allowed the antibody titration in fish immunized against columnaris. The results achieved support a standardized technique that, despite being performed at an experimental level, could be used in the market as a laboratory method to verify the health and/or immunization status of *C. macropomum*.

In our study, it was observed that, on day 0, there was a similarity in antibody titers between groups G1, G3, and G4, while G2 had a greater abundance. Operational factors can influence the accuracy of the test and result in a false positive, such as the use of a polyclonal antibody, which has less specificity compared to a monoclonal antibody and has the capacity to recognize different epitopes, enabling cross-reactivity in the ELISA [58]. The selection of antibodies is necessary for the development of ELISA tests, considering the limitations of monoclonal antibodies and the broader recognition range of polyclonal antibodies. However, these processes require significant expenditure and preparation time [59]. Furthermore, there is also the possibility of the prior exposure of individuals in G2 to *Flavobacterium oreochromis*, since the bacteria are present in the aquatic environment and healthy fish may be infected [60]; however, the disease could manifest itself when associated with predisposing factors such as poor water quality, injuries, and prolonged transportation [5,61]. Similar variations in initial antibody titers have been reported in other fish species [62,63] or in unvaccinated fish maintained under laboratory conditions [64], underscoring the importance of evaluating the dynamics of the immune response rather than focusing solely on baseline levels.

The fish were immunized on day 0 and received a booster on day 21. The increase in antibody abundance between these days, especially in G1 and G2, was relatively low compared to the increase observed between days 21 and 40 (Figure 1). During this period, a safe challenge (day 30) was performed with bacterin to assess the humoral response of *C. macropomum*. The increase in antibody abundance demonstrates that the vaccine booster is an important factor for enhancing fish immunity against *F. oreochromis*, as the absorbance values of groups G1 and G2 remained high until day 60 (higher than the calculated cut-off, Figure 2), with the peak antibody level recorded on day 40 after the first immunization. Our analysis focused on the relative increase in antibody levels within each group over time, rather than directly comparing absolute antibody titers across groups. It is important to note that G2 exhibited higher initial antibody levels compared to the other groups. Nevertheless, the observed increase in antibody levels over time, particularly after the booster dose, suggests an immunological response to vaccination. In contrast, a study evaluating *Rhamdia quelen* vaccinated against *Aeromonas hydrophila* observed that the peak antibody titer occurred on day 21 after vaccination with a single dose [65]. The humoral immunity of *Oreochromis* spp. against TiLV showed a significant increase in antibody levels between day 0 and day 14 after challenge, with levels remaining elevated for up to six weeks. The research evaluated the individual immune response and demonstrated variations in antibody titers between individuals [66]. The interpretation of these results suggests that the pattern of humoral immune response is associated with the fish species and pathogens studied, which is reflected in the processing of immunological analyses and the standardization of the immunoassay.

Since this is the first ELISA developed to detect *C. macropomum* antibodies generated against *F. oreochromis*, there is no gold standard for performing comparative analyses or determining the specificity and sensitivity of the technique. Therefore, we consider the data presented here as preliminary. In future studies, we aim to obtain IgY, particularly from egg yolk, and assess whether it has protective effects (e.g., phagocytosis and bactericidal activity) and its prophylactic and therapeutic potential for *C. macropomum* immunization. Additionally, we plan to perform comparative analyses using highly accurate immunoassays (such as flow cytometry) and conduct experiments with a larger number of immunized animals, thereby providing a more comprehensive characterization of the iELISA described here and validating the technique using samples collected from the field.

## 5. Conclusions

In conclusion, this study demonstrated the potential of anti-*C. macropomum* IgY in recognizing the fish’s humoral immunity, ensuring the reliability of the standardization of the iELISA that was developed. The IgY obtained from hen serum in this study was able to react specifically against epitopes present in the serological material of *Colossoma macropomum* and can be used in the standardized iELISA protocol to detect antibodies from this fish species immunized against *Flavobacterium oreochromis*. The application of the iELISA will enable the analysis of antibody titration and the validation of vaccine protocols in future studies.

## Figures and Tables

**Table 1:** Linear regression analysis with a random effect for absorbance values. G1 was selected as the reference for comparison with G2, G3, and G4. Different letters indicate significant differences between the groups.

Predictors	Estimates	Confidence Interval	*p*-Value
G1—Bacterin + Adjuvant	Ref.	Ref.	^a^
G2—Bacterin	0.01	−0.00–0.02	0.091 ^a^
G3—Adjuvant	−0.05	−0.06–−0.04	<0.001 ^b^
G4—Control	−0.05	−0.06–−0.04	<0.001 ^b^

## Data Availability

The data will be made available on request.
